# Twentieth Century Practice

**Published:** 1897-05

**Authors:** 


### Book Reviews.

TWENTIETH CENTURY PRACTICE. An International Encyclopedia of Modern Medical Science. By leading authorities of Europe and America. Edited by THOMAS L. STEDMAN, M. D., New York City. In twenty volumes. Volume X. Diseases of the Nervous System. New York: William Wood & Co. 1897.

The excuse for such a monumental work is to set forth the present view of the profession on all questions pertaining to medicine. The articles should not represent a given writer's view to the exclusion of other authorities. This being an encyclopedia we look for completeness.

Joseph Collins, of New York, has written most clearly and admirably on diseases of the brain. He has discarded the view that cerebral anemia and hyperemia are clinical entities and has very properly omitted their consideration.

His pages on localisation are up to date and show very clearly the debatable ground is still very large, as the following quotations will show, viz.:

The seat of representation for the trunk muscles is not very well known.

The anatomical limitations put upon the face area by different observers vary considerably.

It is very *probable* that the primary visual center occupies only the medial side of the occipital lobe.

The conclusions of physiologists and of clinicians are not in entire accord as to the cortical localisation of the auditory center.

The seat of the cortical center for smell is not yet satisfactorily determined.

The alleged position of the center for the sense of taste is such that experimental evidence of its existence is difficult to obtain.

Localisations in the white substance of the brain cannot be made with anything approaching the accuracy of that of cortical localisation.

Localisation in the optic thalamus is very uncertain.

These quotations are made to show the fairness of the author. His discussion of diseases of the brain is thoroughly done, yet we must criticise its tendency to assume too much on the intelligence of the reader. Authors should be definite in their directions for treatment. Especially in dosage should directions be explicit in a work of this character. Thus, on page 430, the directions for treatment of syphilitic meningitis include such careless directions: iodide of potassium should be given in large doses. Just what is meant should be stated, that the student may know what the authority he quotes considers a large dose.

Sachs, of New York, discusses brain tumors in fifty well-written pages. He still includes cerebellar tumors in the category of inoperable tumors. He is hopeful that with improved methods of operating, the records of the future will be an improvement on that of the past.

The editor was wise in selecting Féré, of Paris, to write upon hysteria, epilepsy, chorea and the spasmodic neuroses. No better descriptions in the same space could be asked for. The only regret is that the bibliography is almost entirely French. This is unfortunate and needless. American medical literature is too rich to be neglected.

The last 100 pages of the book contain good articles on neurasthenia, by Dana, of New York; disorders of speech, by Pershing, of Denver; disorders of sleep, by Sanger Brown, of Chicago. They are good, but contain nothing noteworthy.

